# Gas concentration prediction in photoacoustic spectroscopy using PSO-EAP-CNN to address correlation degradation

**DOI:** 10.1016/j.pacs.2025.100717

**Published:** 2025-03-28

**Authors:** Zhanshang Su, Pengpeng Wang, Zhengzhuo Li, Yawen Li, Tianxiang Zhao, Yujie Duan, Fupeng Wang, Cunguang Zhu

**Affiliations:** a School of Physical Science and Information Technology, Liaocheng University, Liaocheng 252000, China; b Faculty of Information Science and Engineering, Engineering Research Center of Advanced Marine Physical Instruments and Equipment (Ministry of Education), Optics and Optoelectronics Laboratory (Qingdao Key Laboratory), Ocean University of China, Qingdao 266100, China

**Keywords:** Photoacoustic spectroscopy, Particle swarm optimization, Convolutional neural network, Ensemble augmented prediction

## Abstract

Photoacoustic spectroscopy (PAS) gas detection is frequently compromised by noise-induced correlation degradation, which significantly impacts measurement accuracy. To mitigate this issue, an advanced convolutional neural network (CNN) architecture, termed PSO-EAP-CNN, is proposed, which combines particle swarm optimization (PSO) with an ensemble augmented prediction (EAP) strategy. The proposed framework employs a multi-scale feature extraction mechanism through its convolutional architecture, while simultaneously optimizing network parameters via PSO, thereby achieving accelerated convergence and improved prediction stability. The incorporation of the EAP strategy further enhances the model's robustness and generalization ability under noisy conditions. Experimental results demonstrate significant improvements: compared to baseline CNN, PSO-EAP-CNN reduces MAE by 43.76 %, RMSE by 39.25 %, and MAPE by 51.15 %; compared to ordinary least squares regression, improvements reach 68.55 %, 67.43 %, and 75.21 % respectively. The model runs in only 10 seconds per execution. This work advances PAS-based gas detection, offering enhanced accuracy and noise resilience for practical trace gas analysis.

## 1 Introduction

Gas detection technology plays a critical role in environmental monitoring [1], [2], [3], industrial safety [4], and medical diagnosis [5], [6], particularly in the precise detection of low-concentration gases. Among various detection methods, photoacoustic spectroscopy (PAS) has emerged as a fundamental tool for trace gas analysis [7], [8], [9], [10], combining the photoacoustic effect with laser absorption spectroscopy to achieve high sensitivity, selectivity, rapid response. However, its practical application faces significant challenges from various noise sources that substantially compromise detection accuracy and sensitivity [11]. These noise interferences, including environmental noise, electronic device noise, airflow noise, and background noise [12], [13], [14], [15], particularly affect low-concentration gas detection.

Low-frequency environmental noise typically originates from sound waves with frequencies below 1 kHz and can be attenuated through high-frequency resonant photoacoustic cells and filtering methods; electronic device noise is related to the inherent characteristics of components such as lasers and microphones. Although low-noise components can be selected, complete elimination is difficult. Airflow noise is affected by the sealing of the photoacoustic cell and the airflow velocity. Therefore, improving the sealing performance of the photoacoustic cell and controlling the airflow velocity are necessary to reduce interference. Background noise [16] originates from solid-state photoacoustic effects caused by the incident laser interacting with the window and walls of the photoacoustic cell. Optimizing the materials and structural design [17] of the photoacoustic cell can effectively reduce this noise. Additionally, modulation noise introduced by laser power fluctuations and frequency jitter further reduces the system's signal-to-noise ratio (SNR), so stabilizing the laser's power and frequency is necessary to mitigate its impact [18], [19]. Noise introduces significant distortions and fluctuations in photoacoustic (PA) signals, even for repeated measurements of the same sample, ultimately disrupting the fundamental positive correlation between signal amplitude and gas concentration. This phenomenon, termed noise-induced correlation degradation, can result in lower concentration measurements exhibiting higher signal amplitudes than their higher concentration counterparts. Such noise-induced signal uncertainty presents substantial challenges to conventional calibration methods that rely on establishing amplitude-concentration relationships using reference gases of known concentrations, consequently compromising prediction accuracy.

Recent advancements in PAS have yielded two primary approaches to address noise interference: (1) system performance enhancement through software and hardware optimization for noise suppression, and (2) direct concentration prediction via regression-based feature extraction algorithms. In the domain of software-based solutions, significant progress has been made through advanced signal processing techniques. Neural network-based approaches, including U-shaped network filtering [20] and frequency-domain neural network filters [21], have demonstrated effective noise reduction while preserving critical signal features. Wavelet transform methods have been successfully integrated with differential PAS [22] and combined with sophisticated decomposition algorithms (SSA-VMD-WTD) [23] to enhance signal-to-noise ratio (SNR) in low-concentration detection. On the hardware front, innovative photoacoustic cell designs have emerged, including trapezoid compound ellipsoid resonant cells [24], differential Helmholtz configurations [25], branched photoacoustic cells [26], and planar-spiral spring OMRs [27], which address acoustic instability and reflection noise through advanced geometric optimization and active noise cancellation. Furthermore, open-cell architectures incorporating MEMS technology [28] and dual-tube MEMS-based spectrophones [29] have expanded PAS applicability to rapid gas flow environments and sub-ppb detection scenarios. In terms of quartz tuning forks (QTF), novel four-prong QTF [30], QTF with low resonance frequency [31], and load capacitance matching QTF [32] have further enhanced detection sensitivity. Additionally, compact optical excitation photoacoustic sensors [33] and photoacoustic greenhouse gas sensors [34] have been designed with higher sensitivity and broader applications. These collective advancements in both software and hardware optimization have established a robust foundation for improving PAS detection accuracy and operational reliability.

In contrast to the approach of eliminating noise to enhance system performance, another class of methods involves utilizing regression algorithms to extract features and directly predict gas concentrations. Such approaches have proven effective in addressing spectral overlap and noise, thereby improving prediction accuracy and robustness. For instance, partial least squares regression (PLSR) [35] and multilinear regression with ridge regression [36] have been successfully applied, demonstrating reliable prediction of multi-component gas mixtures under overlapping spectral conditions.

With continuing advancements in computational capabilities, deep learning has emerged as a key research focus in gas concentration prediction. Unlike traditional regression approaches, deep learning [37] autonomously extracts high-dimensional nonlinear features while effectively suppressing noise, thereby significantly improving prediction accuracy. Recent studies have demonstrated this potential through various network architectures: an improved Gaussian-TCN model [38] that outperforms conventional recurrent methods, multi-sensor neural networks [39] achieving precise CO and O₂ detection in complex environments, and residual network frameworks [40] demonstrating robust performance in PAS-based methane retrieval. Collectively, these studies demonstrate deep learning’s capacity to deliver accurate and reliable gas analysis under diverse and challenging conditions. Inspired by these works, we propose a novel deep learning framework integrating convolutional neural networks (CNN) with particle swarm optimization (PSO) and an ensemble augmented prediction (EAP) strategy to mitigate noise-induced correlation degradation in PAS for gas concentration prediction.

The model is trained to automatically learn features directly from the raw waveform, thereby enabling accurate and robust end-to-end concentration predictions, instead of using traditional calibration methods. The main innovations of this work include:

(1) The integration of PSO with CNN, where CNN extracts multi-scale features from the complete waveform of PA signals for gas concentration prediction, while PSO optimizes the neural network parameters to significantly accelerate the model's convergence speed, thus improving the stability and accuracy of predictions under correlation degradation;

(2) The development of an EAP strategy that enhances the model's robustness and generalization ability in addressing noise-induced correlation degradation.

Experimental results demonstrate that the PSO-EAP-CNN model exhibits excellent prediction accuracy and robustness, providing a new method for efficient and precise gas detection in complex working environments, and promoting the application and development of deep learning in the field of gas detection.

## 2 Principles of PAS

PAS operates on the principle of the photoacoustic effect, wherein gas molecules undergo electronic transitions from ground to excited states upon absorbing modulated optical radiation. Through non-radiative relaxation processes, the absorbed energy is converted into thermal energy, inducing periodic temperature fluctuations within the gas medium. These thermal oscillations, synchronized with the light modulation frequency, generate corresponding pressure waves that are detectable as acoustic signals.

Within the photoacoustic cell, a highly sensitive microphone transduces the generated acoustic waves into electrical signals, enabling quantitative gas concentration analysis. The PA signal intensity (*S*) can be mathematically expressed as:

(1) $$S=S_{m}FP_{0}C\alpha (\lambda )$$

where *S*_m_ is the microphone sensitivity, *F* is the cell constant, *P*_0_ is the incident light power, *C* is the gas concentration. The absorption coefficient *α*(*λ*) is determined by the molecular number density *N* and the absorption cross-section *σ*, as expressed below:

(2) α(λ)=N⋅σ

As indicated in Eq. 1, the PA signal intensity (*S*) exhibits a theoretical linear relationship with gas concentration (*C*), which is typically established through calibration procedures for quantitative gas detection. However, practical applications often encounter noise-induced disturbances that degrade this linear correlation, significantly compromising the reliability of conventional calibration methods. To address this limitation, the present study implements a deep learning framework that analyzes comprehensive spectral features of the PA signal, rather than relying solely on amplitude information. This approach establishes an effective nonlinear mapping between the PA signal characteristics and gas concentration, enabling more accurate prediction under noisy conditions.

## 3 PSO-EAP-CNN model

### 3.1 Implementation process

**Fig. 1:**
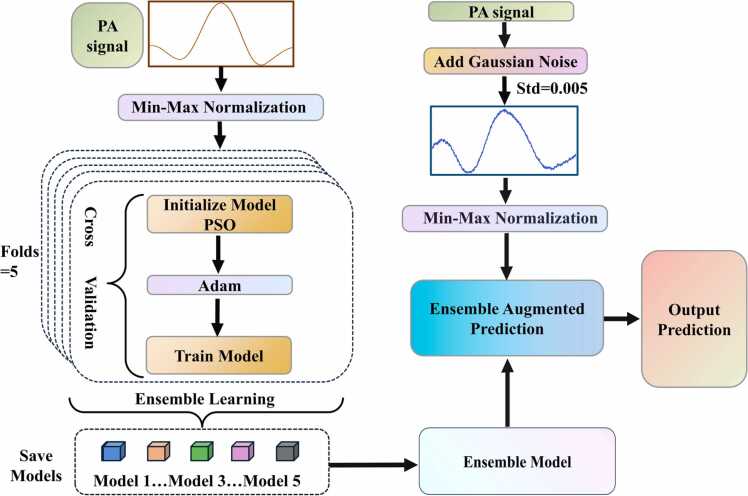
Flowchart for the implementation of the PSO-EAP-CNN model.

The flowchart for the implementation of the PSO-EAP-CNN model is shown in Fig. 1. The entire process consists of four main steps:

Step 1: Data Preprocessing. The PA signals from PAS gas detection system are collected and preprocessed using Min-Max normalization to scale the data to the [0,1] range. This normalization process helps balance feature scales, prevents gradient vanishing or explosion, and accelerates the model's convergence process.

Step 2: Dataset Division and Base Model Construction. Five independent base CNN models are established through five-fold cross-validation. The dataset is randomly split into five subsets (each containing 20 % of the data), with each subset alternately serving as the validation set while the remaining four subsets form the training set, meaning that in each fold, 80 % of the data is used for training and 20 % is used for validation. Through this process, each of the five base models is trained on different combinations of the data, enabling them to learn diverse features and enhancing the ensemble model diversity.

Step 3: Model Optimization and Training. For each fold, an independent CNN model is initialized and optimized. The PSO algorithm is first employed to optimize the model's weights and biases, providing high-quality initial solutions for the subsequent Adam optimizer. During the training process, a dynamic learning rate adjustment strategy is implemented using the StepLR scheduler, with a step size of 500 and a decay factor of 0.1. The model was trained for a total of 1300 epochs, with the initial learning rate set to 0.001. When reaching the 500th epoch, the learning rate was multiplied by the decay factor 0.1, reducing to 0.0001. If training continued to the 1000th epoch, the learning rate decayed further to 0.00001. The larger initial learning rate helps quick convergence, while the reduced learning rate in later stages enables more precise parameter updates.

Step 4: Ensemble Model and Ensemble Augmented Prediction. Due to the diversity in training data and PSO optimization processes, each base model develops distinct initial parameters and optimization paths, leading to complementary learning characteristics. These five base models are then integrated through ensemble learning to create an ensemble model with enhanced generalization ability and prediction accuracy. Finally, to address noise-induced correlation degradation and improve prediction accuracy, an EAP strategy is implemented by adding Gaussian noise to the test dataset and performing multiple predictions with the ensemble model.

### 3.2 Architecture

**Fig. 2:**
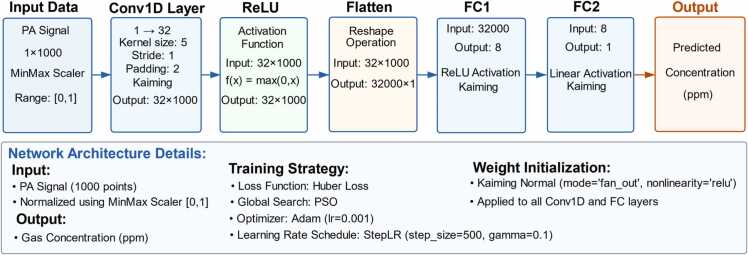
Architecture of CNN model.

The CNN architecture is shown in Fig. 2. It consists of an input layer, a one-dimensional convolutional (Conv1D) layer, a ReLU activation layer, a flatten layer, and two fully connected (FC) layers (FC1 and FC2).

The PA signals collected in experiments are used as the input to the neural network, with each input sample featuring a vector dimension of 1000, and all signals are normalized before being fed into the network. The Conv1D layer maps the input signal from a channel to 32 feature channels using 32 convolution kernels with a kernel size of 5 and a stride of 1, and employs padding to maintain an output dimension of 32 × 1000. A ReLU activation function follows the Conv1D layer, enhancing the network's feature expression capability by introducing nonlinearity. After the Conv1D and ReLU layers, the signal is flattened into a one-dimensional vector of size 32000 × 1 to meet the input requirements of the subsequent fully connected layers. The FC1 compresses the flattened 32000-dimensional feature vector to 8 dimensions to extract high-level features. The ReLU activation function is also employed in this layer to augment the model's capability for nonlinear representation. Finally, FC2 uses a linear activation function to map the 8-dimensional feature vector to a single output node, directly predicting the gas concentration value. To ensure the stability and convergence efficiency of network training, the weights of all convolutional and fully connected layers are initialized using Kaiming initialization. For the training strategy, Huber Loss is employed as the loss function to enhance robustness against outliers in PA signals. PSO is utilized for global search, with Adam (lr=0.001) as the network optimizer, along with a StepLR dynamic learning rate scheduling strategy to balance the convergence speed and stability of training.

To address the challenges of concentration prediction caused by correlation degradation in PAS gas detection systems, we propose an integrated optimization strategy combining PSO and EAP. Specifically, the synergy between CNN and PSO establishes a robust feature extraction framework: CNN extracts comprehensive features from PA signals, while PSO significantly reduces the risk of converging to local optima during training, thereby enhancing both feature extraction capabilities and training stability. Furthermore, the introduction of the EAP strategy effectively reduces prediction variance and improves the model's robustness against noise and outliers. This dual-optimization strategy effectively addresses correlation degradation in PAS-based gas concentration measurements, thereby enabling accurate gas concentration prediction. Subsequently, both the PSO and EAP optimization strategies will be discussed in detail.

### 3.3 Optimization strategies

#### 3.3.1 PSO optimization strategy

By leveraging the PSO algorithm to optimize CNN parameters, the proposed PSO-EAP-CNN model achieves remarkable improvements in stability and generalization ability, offering a robust solution for accurate gas concentration prediction in PAS systems. As a heuristic global optimization algorithm, PSO emulates the collaborative search behavior of particles in a multi-dimensional search space, effectively avoiding local optima and is particularly suitable for high-dimensional, non-convex optimization problems.

**Fig. 3:**
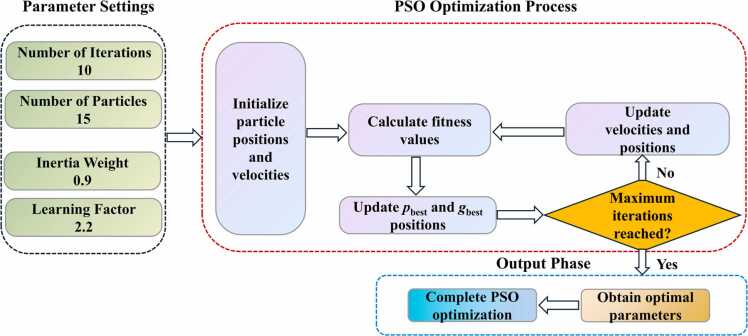
Flowchart of PSO algorithm implementation.

The proposed PSO implementation consists of three sequential stages: parameter settings, optimization process, and output phase (Fig. 3). Prior to the optimization process, four key parameters are configured: number of iterations (10), number of particles (15), inertia weight (0.9), and learning factor (2.2). These parameters are chosen to provide an appropriate balance between the algorithm’s exploration and exploitation during the optimization process.

In this study, PSO mainly optimizes the training parameters of the CNN model, including the weights and biases of both convolutional and fully connected layers. Specifically, each particle represents a complete set of model parameters, including the weights (32 ×1 ×5) and biases (32) of the Conv1D layer, as well as the weights (32000 ×8) and biases (8) of FC1, and the weights (8 ×1) and biases (1) of FC2.

The optimization process begins with particle initialization, where particle positions and velocities are randomly initialized in the parameter space. Subsequently, the algorithm enters an iterative optimization loop consisting of three key steps：

Step 1: Fitness value calculation for each particle using the Huber loss function;

Step 2: Update of personal best (*p*_best_) and global best (*g*_best_) positions based on fitness evaluations;

Step 3: Velocity and position updates according to:

(3) $$v_{i}^{t+1}=w\cdot v_{i}^{t}+c_{1}\cdot r_{1}\cdot \left(p_{best}-x_{i}^{t}\right)+c_{2}\cdot r_{2}\cdot \left(g_{best}-x_{i}^{t}\right)$$

(4) $$x_{i}^{t+1}=x_{i}^{t}+v_{i}^{t+1}$$

where *w* is the inertia weight; *c*_1_ and *c*_2_ are the individual and social learning factors, respectively; *r*_1_ and *r*_2_ are random numbers in the range [0,1], used to increase the randomness of the search process; *p*_best_ is the personal best position of the particle; and *g*_best_ is the global best position of the swarm. Through multiple iterations, the particles' velocities and positions are continuously updated, gradually approaching the optimal solution in the loss function space.

The fitness evaluation employs the Huber loss function, which exhibits unique piecewise characteristics:

(5) $$L_{\delta }\left(a\right)=\{\begin{array}{ll} \frac{1}{2}a^{2}, & \mathrm{if}\left|a\right|\leq \delta \\ \delta \left(\left|a\right|-\frac{1}{2}\delta \right), & \mathrm{otherwise} \end{array}$$

where,*α* is the error between the predicted value and the true value; *δ* is a threshold.

Compared to the traditional Mean Squared Error loss function, the Huber loss function demonstrates superior robustness against outliers, making it particularly suitable for handling data in complex noisy environments. It behaves similarly to MSE in regions with small errors while adopting linear growth once the error exceeds a threshold, thereby effectively reducing the impact of noise-induced anomalies in PA signals. This design not only enhances the model's robustness to noisy data but also helps prevent overfitting issues.

The iterative optimization process continues until the maximum number of iterations is reached, at which point the algorithm enters the output phase. During this final phase, the optimal parameters are obtained based on the *g*_best_ position achieved throughout the optimization process, completing the PSO optimization procedure. This systematic PSO-based optimization approach not only ensures the stability of feature extraction but also enhances the CNN model's PA signal feature extraction capability.

#### 3.3.2 Ensemble augmented prediction strategy

**Fig. 4:**
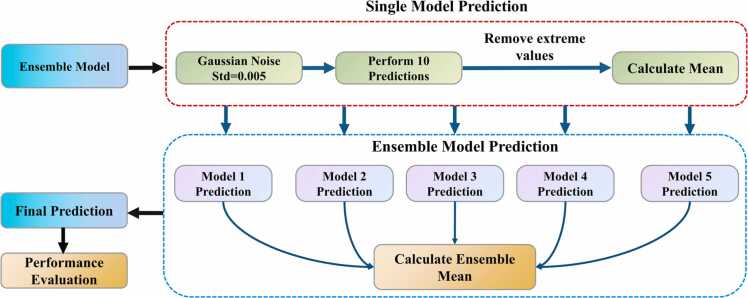
Flowchart of EAP strategy implementation.

This study introduces the EAP strategy to address prediction variance and enhance robustness in gas concentration estimation under correlation degradation conditions. The proposed EAP strategy establishes a standardized prediction procedure for each model, comprising Gaussian noise injection (std=0.005), multiple predictions, extreme value elimination, and statistical averaging, as illustrated in Fig. 4.

The specific implementation of this strategy consists of two main parts. In the first part, each model in the ensemble follows a standardized prediction procedure. Considering the problems of noise-induced correlation degradation in PAS sensor measurements, random Gaussian noise with a standard deviation of 0.005 is added to the test data. Each normalized test sample undergoes ten predictions with random noise perturbations. To extract more robust estimates, extreme values from these predictions are removed, and the remaining predictions are averaged to form a single model's output.

In the second part, this standardized prediction procedure is systematically applied to the ensemble models derived through cross-validation and ensemble learning techniques. Each model independently processes the test data through the same noise perturbation and statistical processing steps, resulting in five refined predictions. These predictions are then averaged to generate the final prediction, which effectively combines the unique strengths of each model while minimizing individual model biases.

The proposed EAP strategy demonstrates significant improvements in model prediction reliability and accuracy under correlation degradation. Compared to traditional calibration methods, the EAP strategy not only minimizes prediction variance but also effectively utilizes the complementary feature extraction capabilities of different models, thereby providing a robust and reliable prediction framework for PAS gas concentration detection systems.

### 3.4 Experimental system and data collection

**Fig. 5:**
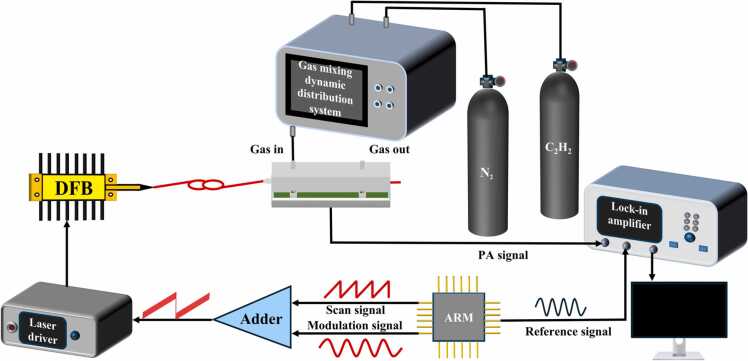
Experimental diagram of the PAS gas detection system.

An experimental PAS gas detection system was developed to validate the performance of the proposed PSO-EAP-CNN prediction model. The system, illustrated in Fig. 5, was specifically designed for precise acquisition of photoacoustic signals across varying gas concentrations, generating a comprehensive dataset for model training and validation.

Using a gas dynamic distribution system (FD-HQ04, Suzhou Friends Experimental Equipment Co., Ltd., China), nitrogen and acetylene were mixed in varying proportions and delivered to a compact photoacoustic (PA) cell with an inner volume of 0.5 mL and a resonance frequency of 4579 Hz, based on 3D-printed differential Helmholtz resonators [41]. A signal generator (FY8300S, FeelElec Technology Co., Ltd., China) was used to generate three distinct waveform signals: a 1 Hz sawtooth wave (CH1), a 2.29 kHz sine wave (CH2), and a 4.58 kHz sine wave (CH3). These signals were designated to modulate the laser's frequency and intensity. The laser driver (M-DFB-GP02, Wuhan 69 Sensing Technology Co., Ltd., China) provided 10-mW of optical power to a 14-pin butterfly-packaged DFB-LD (DFB-1532-F-N, Sichuan Tengguang Electronics and Technology Co., Ltd., China) operating near a wavelength of 1532.68 nm. As the emitted laser traversed the PA cell, interactions between the laser light and gas molecules occurred. The resulting PA signal was subsequently detected by a microphone integrated within the PA cell. This microphone converted the PA signal into an electrical signal, which was transmitted to the lock-in amplifier for the second harmonic signal (2 *f*) extraction.

**Fig. 6:**
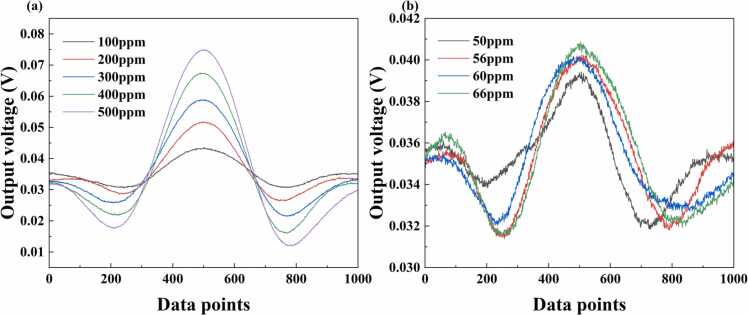
PA signal characteristics at different concentration ranges.

Three sets of PAS data were collected within the concentration range of 40–500 ppm, with two sets used for model training and one set for testing. Fig. 6 illustrates the characteristics of PA signals at different concentration ranges. Fig. 6(a) shows a portion of PA signals in the higher concentration range (100–500 ppm), where a distinct positive correlation can be observed between signal amplitude and gas concentration, with PA signal amplitudes showing clear enhancement as the gas concentration increases. Fig. 6(b) depicts PA signals in a part of the low concentration range (50–66 ppm), where noise-induced signal distortion and fluctuation lead to correlation degradation between signal amplitude and gas concentration. These effects are particularly pronounced at lower concentrations due to reduced SNR, which poses significant challenges for traditional calibration methods.

## 4 Experimental validation and performance analysis

### 4.1 Model validation and parameter optimization for PSO-EAP-CNN

This section first validates the optimization performance of the PSO and EAP strategies individually, determining their optimal parameters. Then, the overall performance of the PSO-EAP-CNN model is evaluated for gas concentration prediction. For a comprehensive evaluation, the test set covered concentrations from 40–500 ppm, with sampling intervals of 10 ppm in the 40–150 ppm range and 20 ppm in the 150–500 ppm range. All experiments were conducted on an NVIDIA GeForce RTX 4060 GPU with Python 3.9.19 and PyTorch 2.0.0 as the deep learning framework.

To comprehensively evaluate the model's prediction performance, this study utilizes four metrics: mean absolute error (MAE), root mean square error (RMSE), mean absolute percentage error (MAPE), and the coefficient of determination (R²). The calculation formulas for these metrics are as follows:

(6) $$MAE=\frac{1}{n}\sum_{i=1}^{n}|y_{i}-{\hat{y}}_{i}|$$

(7) $$RMSE=\sqrt{\frac{1}{n}\sum_{i=1}^{n}{\left(y_{i}-{\hat{y}}_{i}\right)}^{2}}$$

(8) $$MAPE=\frac{1}{n}\sum_{i=1}^{n}|\frac{y_{i}-{\hat{y}}_{i}}{y_{i}}|\times 100\%$$

(9) $$R^{2}=1-\frac{\sum_{i=1}^{n}{\left(y_{i}-{\hat{y}}_{i}\right)}^{2}}{\sum_{i=1}^{n}{\left(y_{i}-\bar{y}\right)}^{2}}$$

where *y*_i_ is the actual concentration value, *ŷ*_i_ is the model's predicted value, *ȳ* is the average actual concentration value, and *n* is the number of samples.

**Fig. 7:**
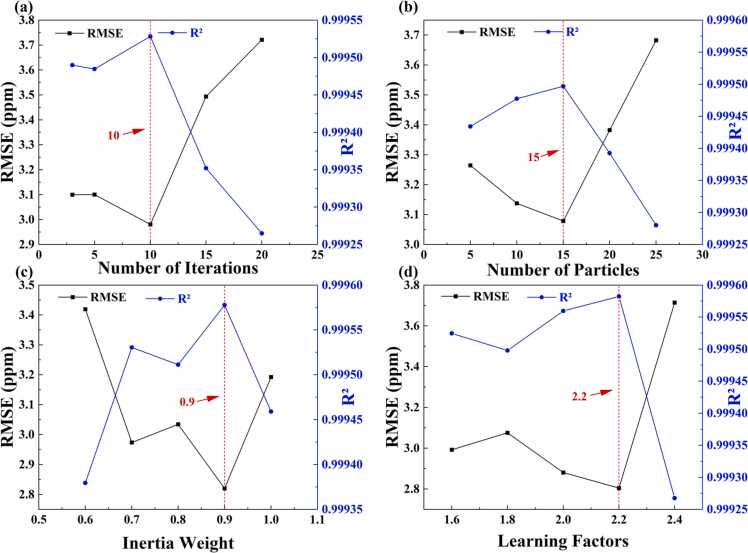
Comprehensive optimization of PSO parameters.

First, we validated the effectiveness of the PSO optimization strategy and determined its optimal parameters. To achieve optimal predictive performance, we conducted a systematic analysis and optimization of the four key parameters in the PSO optimization strategy: number of iterations, number of particles, inertia weight, and learning factors. Fig. 7 illustrates the impact of these parameters on model performance, where the x-axis represents the parameter values and the y-axis indicates RMSE and R² values. Fig. 7(a) demonstrates that when the number of iterations is set to 10, the model achieves optimal RMSE and R² values of 2.981 ppm and 0.99953, respectively. Further increasing the number of iterations leads to a decline in performance, which may be attributed to overfitting. Fig. 7(b) indicates that the model performs best with 15 particles, achieving RMSE and R² values of 3.079 ppm and 0.99950, respectively, as an excessive or insufficient number of particles may affect the model's convergence. Fig. 7(c) demonstrates that with an inertia weight of 0.9, the model exhibits optimal global search capability, with RMSE decreasing to a minimum of 2.821 ppm and R² reaching a maximum of 0.99958. Fig. 7(d) shows that the model achieves optimal performance when the learning factor is 2.2, with RMSE and R² values of 2.804 ppm and 0.99958, respectively. Through extensive experimental validation, we identified the optimal combination of parameters: 10 iterations, 15 particles, an inertia weight of 0.9, and a learning factor of 2.2. The selection of this optimized parameter set not only ensures an efficient search by the PSO optimization strategy within the CNN parameter space but also effectively avoids local optima issues, thereby providing a reliable parameter foundation for the PSO-EAP-CNN model in PAS gas concentration prediction.

**Fig. 8:**
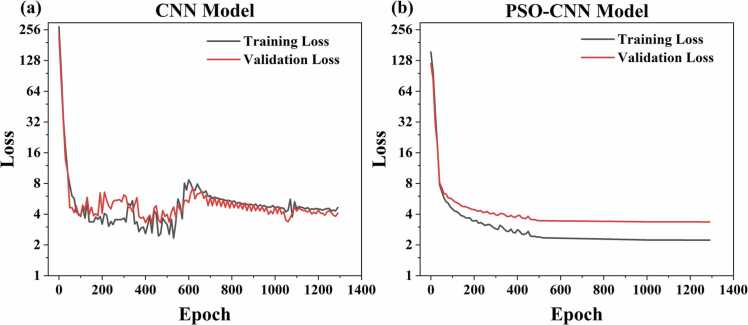
Comparison of training loss curves between PSO-CNN and CNN.

Fig. 8 presents a comparison of training loss curves before and after PSO optimization, demonstrating three major improvements: First, the convergence speed increased significantly, with loss values rapidly decreasing from 128 to 4 within the first 200 epochs; Second, after 600 epochs, both training and validation losses stabilized at approximately 2–4, indicating the model possesses good generalization ability. Third, unlike the basic CNN model which exhibits significant fluctuations between 4 and 8 during epochs 200–1000, the optimized model maintained stable convergence. Moreover, the runtime of our program is maintained at approximately 10 seconds, demonstrating that the PSO optimization strategy maintains high computational efficiency.

Based on the evaluation metrics on the test set, the performance of PSO-CNN with PSO optimization has been significantly improved. MAE was reduced by 28.01 % (from 3.677 ppm to 2.647 ppm); RMSE decreased by 15.50 % (from 4.484 ppm to 3.789 ppm); MAPE saw a 27.63 % reduction (from 2.092 % to 1.514 %); and the R² value improved slightly from 0.9988 to 0.9992. The results demonstrate that PSO optimization effectively enhances the model's prediction accuracy and generalization ability in PAS gas detection systems.

**Fig. 9:**
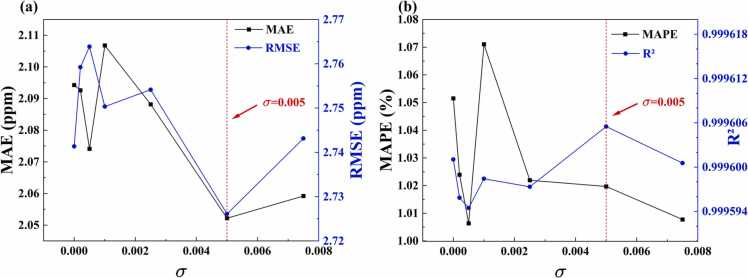
Evaluation under different noise disturbances.

Next, we validated the effectiveness of the EAP strategy and determined its optimal parameters. The EAP strategy introduces controlled Gaussian noise during prediction to reduce the prediction variance of individual models and enhance ensemble model generalization ability, particularly improving prediction accuracy under correlation degradation conditions. To determine the optimal noise configuration, we systematically analyzed the impact of various noise parameters on model performance. As illustrated in Fig. 9, when there is no noise (*σ*=0), the model's MAE is 2.094 ppm, RMSE is 2.741 ppm, MAPE is 1.052 %, and R² is 0.999601. As the noise level increases, the model's performance demonstrates significant changes. When *σ* increases to 0.005, the model's performance attains optimal levels across multiple metrics: MAE decreases to 2.052 ppm, RMSE decreases to 2.726 ppm, and R² increases to 0.999606, indicating that moderate noise enhances the model's generalization ability. Notably, MAPE attains its lowest value of 1.006 % at *σ*= 0.0005 and remains at a relatively low level of 1.020 % at *σ*= 0.005, demonstrating good stability. Further analysis indicates that when *σ* > 0.005, the evaluation metrics begin to deteriorate markedly: MAE and RMSE gradually increase to 2.059 ppm and 2.743 ppm, respectively. This phenomenon indicates that excessive noise intensity reduces the model's prediction accuracy. Considering the performance of all evaluation metrics, *σ*= 0.005 is identified as the optimal noise parameter configuration for this model in complex noise environments, effectively improving the accuracy of PAS gas concentration prediction.

**Fig. 10:**
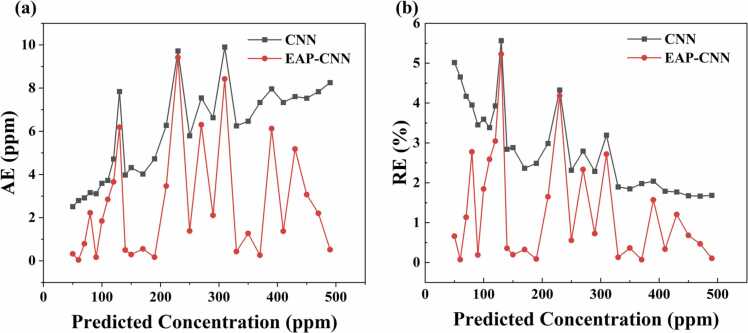
Error distribution of AE and RE for CNN and EAP-CNN.

The effectiveness of the EAP strategy in reducing prediction errors can be visually demonstrated in Fig. 10. Across all concentration levels in the test set, the absolute error (AE) and relative error (RE) curves of EAP-CNN with EAP optimization are consistently lower than those of the basic CNN. This significant reduction and distribution of errors fully demonstrate the effectiveness of the EAP strategy in improving prediction stability. EAP optimization resulted in significant improvements: compared to the basic CNN, the EAP-CNN's MAE decreased from 3.677 ppm to 2.583 ppm, a reduction of 29.75 %; RMSE decreased from 4.484 ppm to 3.720 ppm, a reduction of 17.04 %; MAPE decreased from 2.092 % to 1.282 %, a reduction of 38.72 %; and the R² value increased from 0.9988 to 0.9993. The comprehensive improvement of these metrics fully validates the effectiveness of the EAP strategy. This optimization effect is mainly attributed to the noise augmentation and ensemble strategies of EAP, which significantly enhance the model’s adaptability to the correlation degradation of PA signals.

**Fig. 11:**
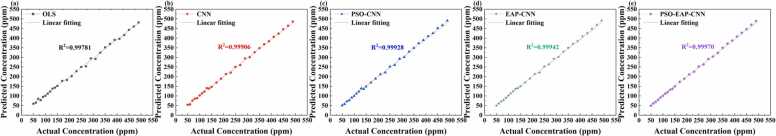
Comparison of R² values between traditional calibration method (OLS) and models with different optimization strategies. (a) OLS, (b) CNN, (c) PSO-CNN, (d) EAP-CNN, (e) PSO-EAP-CNN.

Finally, we evaluated the overall performance of the PSO-EAP-CNN model that incorporates both PSO and EAP optimization strategies. To further validate the generalization ability of our proposed model, we conducted a systematic analysis of prediction performance across the concentration range of 50–500 ppm. Fig. 11 illustrates the fitting performance of different models between actual and predicted concentrations. The ordinary least squares (OLS) method serves as a baseline with an R²= 0.99781 (Fig. 11a). The PSO-EAP-CNN model demonstrates superior fitting performance across the entire concentration range, with an R²= 0.99970 (Fig. 11e) significantly outperforming other models, indicating higher prediction accuracy and better model generalization ability. The R² values for PSO-CNN and EAP-CNN are 0.99928 (Fig. 11c) and 0.99942 (Fig. 11d), respectively, both of which exceed the R² value of 0.99906 (Fig. 11b) for the basic CNN model. All neural network-based models demonstrate improved performance compared to the traditional OLS method. However, among all neural network approaches, the PSO-EAP-CNN achieves the highest R² value, which strongly validates the synergistic enhancement effect of combining the PSO and EAP strategies.

**Fig. 12:**
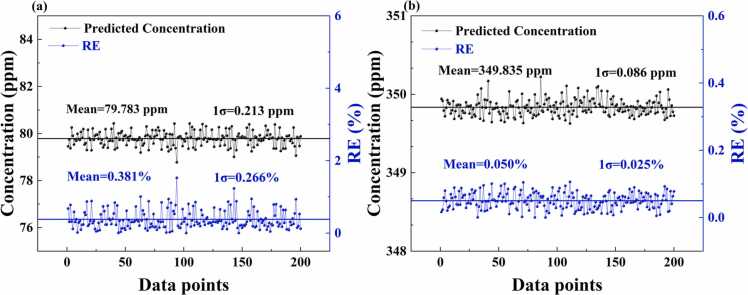
(a) and (b) show the concentration results and RE distribution for 200 consecutive predictions at 80 ppm and 350 ppm, respectively.

Regarding the stability of the model, Fig. 12(a) and (b) show the concentration results and RE distribution for 200 consecutive predictions at concentrations of 80 ppm and 350 ppm, respectively. At the concentration of 80 ppm, the PSO-EAP-CNN model achieved an average predicted concentration of 79.783 ppm (standard deviation: 0.213 ppm) and an average relative error of 0.381 % (standard deviation: 0.266 %). At the concentration of 350 ppm, the average predicted concentration was 349.835 ppm (standard deviation: 0.086 ppm), with an average relative error of 0.050 % (standard deviation: 0.025 %). These results indicate that the PSO-EAP-CNN model not only demonstrates excellent prediction accuracy and robustness across a wide concentration range, but also exhibits high stability across multiple consecutive predictions, further validating its reliability and consistency in key concentration regions.

Notably, as shown in Table 1, the combination of PSO and EAP strategies (PSO-EAP-CNN) demonstrated even more remarkable improvements in prediction accuracy. Compared with PSO-CNN, the PSO-EAP-CNN reduced MAE by 21.88 %, RMSE by 28.09 %, and MAPE by 32.50 %. Similarly, when compared to EAP-CNN, the PSO-EAP-CNN achieved reductions of 19.97 %, 26.76 %, and 20.28 % in MAE, RMSE, and MAPE, respectively. This superior performance can be primarily attributed to the synergistic effect of combining PSO and EAP strategies: PSO optimizes the network’s weights and biases, thereby enhancing feature extraction capabilities and improving prediction stability. Meanwhile, EAP reduces the variance of single model prediction to improve robustness and prediction accuracy. The integration of these complementary approaches significantly improves the prediction accuracy under correlation degradation.

**Table 1:** Performance evaluation before and after optimization with PSO and EAP strategies.

Models	MAE/ppm	RMSE/ppm	MAPE/%	R^2^
PSO-EAP-CNN	2.068	2.724	1.022	0.9996
CNN	3.677	4.484	2.092	0.9988
PSO-CNN	2.647	3.789	1.514	0.9992
EAP-CNN	2.583	3.720	1.282	0.9993

**Fig. 13:**
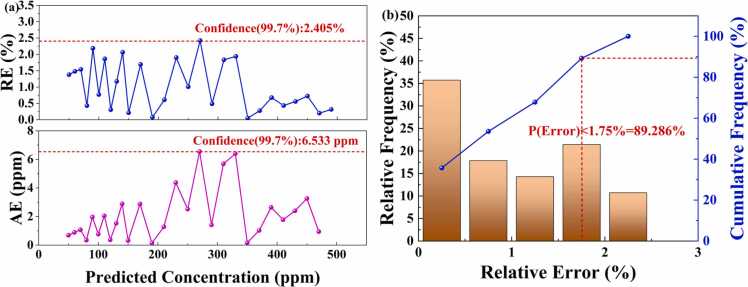
(a) Error distribution for 50–500 ppm. (b) Relative error probability distribution within the concentration range of 50–500 ppm.

To thoroughly evaluate the performance of the PSO-EAP-CNN model in practical application scenarios, we investigated both the error distribution and relative error probability distribution on the test set. Fig. 13(a) illustrates the variations in RE and AE between the model's predicted concentrations and actual concentrations. The results indicate that within a 99.7 % confidence interval, the RE is 2.405 % and the AE is 6.533 ppm, demonstrating that the model maintains high accuracy and uniformly distributed errors across a wide concentration range. Fig. 13(b) presents the relative frequency distribution and cumulative frequency distribution of errors, with analysis showing that 89.286 % of the predicted relative errors are below 1.75 %, and most errors are concentrated below 1 %, fully reflecting the model's excellent error control capability and high-precision characteristics.

### 4.2 Comparison with other models

To evaluate the effectiveness of our proposed method, the PSO-EAP-CNN model was compared with several commonly used predictive models including CNN, LSTM, and OLS. Table 2 presents the regression evaluation metrics for each model based on the overall performance assessment of the test set. The PSO-EAP-CNN model outperformed other models across the three metrics MAE, RMSE, and MAPE, achieving values of 2.068 ppm, 2.724 ppm, and 1.022 %, respectively. Relative to the CNN model, reductions of approximately 43.76 % (MAE), 39.25 % (RMSE), and 51.15 % (MAPE) were observed. When benchmarked against the LSTM, the corresponding decreases reached 66.23 %, 63.29 %, and 73.12 %. In comparison with the OLS approach, the improvements were even more pronounced, with MAE, RMSE, and MAPE reduced by 68.55 %, 67.43 %, and 75.21 %, respectively. Additionally, the R² value of PSO-EAP-CNN reached 0.9996, which is closest to 1, indicating that the model exhibits excellent fitting ability and generalization ability. In contrast, CNN, LSTM, and OLS models exhibited significantly higher errors and slightly lower R² values, revealing their limitations in complex data environments.

**Table 2:** Performance of different models on the test set.

Models	MAE/ppm	RMSE/ppm	MAPE/%	R^2^
CNN	3.677	4.484	2.092	0.9988
LSTM	6.122	7.420	3.802	0.9967
OLS	6.574	8.363	4.123	0.9963
PSO-EAP-CNN	2.068	2.724	1.022	0.9996

**Fig. 14:**
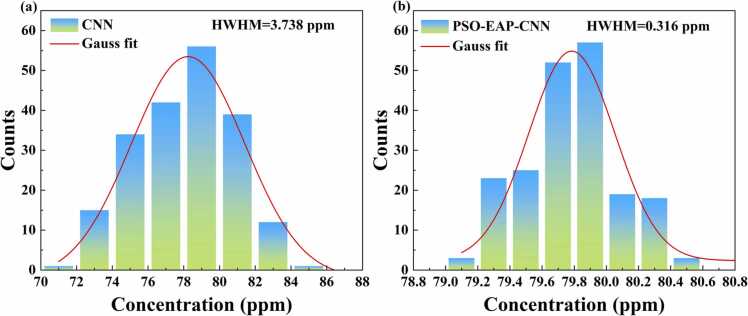
Statistical histograms of concentration distribution for the CNN and PSO-EAP-CNN models.

To further validate the distributional characteristics of the model predictions, a Gaussian fitting analysis was conducted on the prediction results. Among the four methods, the PSO-EAP-CNN model demonstrated the best performance, followed by the CNN model. Therefore, we selected these two models for comparative analysis of their prediction results. As shown in Fig. 14(a), the distribution of predicted concentration values by the CNN model is relatively dispersed, with the half-width at half-maximum (HWHM) of the fitting curve reaching approximately 3.738 ppm. In contrast, as shown in Fig. 14(b), the distribution of the prediction results from the PSO-EAP-CNN model is markedly more concentrated. The HWHM of its Gaussian fitting curve is only about 0.316 ppm, significantly lower than that of the CNN model.

## 5 Conclusion

This study proposes a PSO-EAP-CNN model to solve the correlation degradation of photoacoustic spectroscopy and achieve accurate gas concentration prediction under complex noise environments. The model can directly process experimental data with noise from PAS systems and effectively prevent overfitting by using an independent test set, ensuring the model's strong generalization ability. Experimental results demonstrate that the PSO-EAP-CNN model significantly outperforms the basic CNN and OLS models across all metrics, with MAE, RMSE, and MAPE reduced by 43.76 %, 39.25 %, and 51.15 % compared to CNN, and by 68.55 %, 67.43 %, and 75.21 % compared to OLS, respectively. The model demonstrates superior fitting performance across the entire concentration range, with an R² of 0.9997, significantly outperforming other models, indicating higher prediction accuracy and better model generalization ability. The probability of the relative error being less than 1.75 % is as high as 89.286 %. Gaussian fitting analysis further validates the model's prediction stability, with the PSO-EAP-CNN model achieving a HWHM of only 0.316 ppm. Notably, the PSO-EAP-CNN model's runtime is maintained at around 10 seconds, demonstrating its advantage in efficiently handling complex data environments. In conclusion, the PSO-EAP-CNN model achieves efficient and high-precision gas concentration prediction in high-noise environments, demonstrating strong application potential. Future research will focus on further optimizing the model structure, improving training accuracy, and expanding the model's applicability in various complex environments to promote its widespread use in practical applications.

## CRediT authorship contribution statement

**Zhanshang Su:** Writing – original draft, Methodology, Investigation. **Pengpeng Wang:** Writing – review & editing, Supervision, Project administration, Funding acquisition. **Zhengzhuo Li:** Methodology, Formal analysis. **Yawen Li:** Validation. **Tianxiang Zhao:** Validation. **Yujie Duan:** Validation. **Fupeng Wang:** Writing – review & editing, Supervision, Project administration. **Cunguang Zhu:** Writing – review & editing, Supervision, Project administration, Funding acquisition.

## Declaration of Competing Interest

The authors declare that they have no known competing financial interests or personal relationships that could have appeared to influence the work reported in this paper.

## Data Availability

Data will be made available on request.
